# Functional Neuromyofascial Activity: Interprofessional Assessment to Inform Person-Centered Participative Care—An Osteopathic Perspective

**DOI:** 10.3390/healthcare11212886

**Published:** 2023-11-02

**Authors:** Francesca Baroni, Robert Schleip, Lorenzo Arcuri, Giacomo Consorti, Giandomenico D’Alessandro, Rafael Zegarra-Parodi, Anna Maria Vitali, Marco Tramontano, Christian Lunghi

**Affiliations:** 1 BMS Formation, 75116 Paris, France; francesca@bms-formation.com (F.B.); christian@bms-formation.com (C.L.); 2 Osteopatia Lunghi-Baroni Private Practice, 00146 Rome, Italy; 3 Department of Sport and Health Sciences, Conservative and Rehabilitative Orthopedics, Technical University of Munich, 80333 Munich, Germany; robert.schleip@tum.de; 4 Clinical-Based Human Research Department, Foundation COME Collaboration, 65121 Pescara, Italy; 5 Education Department of Osteopathy, Istituto Superiore di Osteopatia, 20126 Milan, Italy; 6 Centre pour l’Etude, la Recherche et la Diffusion Ostéopathiques “C.E.R.D.O.”, 00199 Rome, Italy; 7 Fisicamente Formazione Private Practice, 00192 Rome, Italy; 8 Department of Biomedical and Neuromotor Sciences (DIBINEM), University of Bologna, 40126 Bologna, Italy; 9 Unit of Occupational Medicine, IRCCS Azienda Ospedaliero, Universitaria di Bologna, 40126 Bologna, Italy

**Keywords:** musculoskeletal physiological phenomena, movement, motion, manipulation, osteopathic, exercise movement techniques, exercise therapy, mind–body therapies, bodyworks

## Abstract

**Introduction**: Health professionals and bodyworkers may be pivotal in promoting prevention programs, providing tailored advice and guidance to patients’ adherence to self-care strategies, such as physical activity. Contemporary evidence encourages manual therapists to involve patients in decision-making and treatment procedures integrating passive and active approaches in treatment planning. This manuscript provides a definition and applications of neuromyofascial movement patterns, discusses the significance of functional assessment, and gives an example of clinical applications in the osteopathic field to highlight how this assessment can promote interdisciplinarity. **Methods**: The reporting framework used in the current manuscript followed guidelines for writing a commentary. **Results**: The manuscript highlights the crucial role that the neuromyofascial system plays in human movement and overall well-being and the importance of a functional neuromyofascial activity assessment in the context of person-centered participative care. **Conclusions**: Understanding individual neuromyofascial patterns could help healthcare practitioners, movement specialists, and bodyworkers in tailoring treatment plans, meeting patients’ unique needs, and promoting a more effective personalized approach to care. The current perspective could spark debates within the professional community and provide a research roadmap for developing an evidence-informed interprofessional framework.

## 1. Introduction

Contemporary global guidelines highlight the importance of regularly undertaking physical activity and provide recommendations for general and specific populations, i.e., pregnant and postpartum women and people with chronic conditions or disabilities [1]. Health professionals involved in primary, secondary, or tertiary prevention programs, allied health workers, and exercise professionals are considered key users to provide tailored advice and guidance to patients [1]. The Guidelines on Physical Activity and Sedentary Behaviour [1] and Interventions for the Management of Acute and Chronic Low Back Pain [2] encourage multimodal, biopsychosocial approaches, combining active and passive approaches with education processes, for patients with chronic musculoskeletal pain [2,3]. The health needs of the contemporary era require us to involve patients in decision-making processes and treatments [4,5]. According to person-centered care principles for musculoskeletal pain, there is a need to improve the therapeutic alliance and to establish meaningful connections with the patient/participant by using the body, and related movement, as a pivot point [4]. Manual therapies can be useful as part of overall management to support a patient’s specific daily activities: physiotherapy [6], chiropractic [7], and osteopathy [8]; providing a combination of manipulative strategies and assisted exercise can supply the movement challenges necessary for optimal recovery [9]. The patient recovery process can be optimized by co-creating based on individual patient environments and considering self-care as a dominant component in process management. Individuals’ actions within their environment are key to recovery [9]. In the past years, foundational motion patterns and building blocks of human actions were proposed as re-educative strategies for human movement: for example, instinctive sleep postures [10], primal movement patterns [11], archetypal postures [12], fetal movements [13], and senior fitness [14] have been proposed as fundamental motion patterns for all human health behaviors. Moreover, different study groups have developed approaches for the analysis of movement patterns as well as rehabilitative approaches to optimize the movement system [15,16,17,18]. The mentioned approaches are based, in part, on the assumption that ideal postural patterns are defined using developmental kinesiology and maintained using core stabilization [15] and on the idea that identifiable deficits in symmetries of movement patterns increase susceptibility to injury [16,17,18]. There is a need to move away from a classical biomechanical vision of health and disease and define an updated clinical tool to evaluate and treat movement patterns [9]. Within the field of manual therapies, in recent years a shift was proposed away from traditional dogmas that associated health with an ideal posture [8]. In fact, a new perspective, emphasizing the importance of considering postural control in terms of an individual’s ability to adapt to their current allostatic load was shared. This adaptation relies on the dynamic interplay of exteroceptive, proprioceptive, and interoceptive perceptions integrated at a neuromyofascial level during musculoskeletal functional activities and coordinated movement [8]. The contemporary knowledge concerning the role of the fascial system represents a cornerstone of the transition from the traditional biomechanical model to a neuromyofascial perspective of human movement and postural changes [8].

Osteopathic principles refer to the musculoskeletal system and its movement as central elements for body regulation systems, from postural control to neural processes, metabolism, circulatory and respiratory capacity, and psychic reactivity [19]. Osteopathy is considered a whole-body patient-centered intervention mainly focused on sustaining a person’s health through touch-based approaches, therapeutic alliance, and education [20] to enhance patients’ adherence to self-care strategies, such as physical activity, exercise, lifestyle, and diet [21,22,23]. 

Osteopathic tenets and theoretical models are experiencing a conceptual revolution; on the one hand, the osteopathic field is being renewed under the umbrella of constructs shared with other healthcare professions, while on the other hand, contact is being maintained with renewed traditional principles that confer the unique position of promoting a scientific model of holistic care [24]. Despite a recent study protocol for clinical trials proposed to evaluate the effects of osteopathic manipulative treatment associated with pain education and clinical hypnosis in individuals with chronic low back pain [25], the linkage between hands-on (i.e., manipulative therapy) and hands-off approaches (i.e., exercise and self-help) is not well established in the community of practice [22]. Thus, curricular training, as well as clinical practice and research conducted in the osteopathic field, are still too focused on passive approaches and not inclined to promote participatory approaches for the patient [26]. Although the movement of the musculoskeletal system is highly regarded in the osteopathic field, there is currently no shared evaluation model. To the best of our knowledge, no formal approach has been developed in the osteopathic field for evaluating functional movement and a person’s capacity to move with good agency and confidence. We hypothesize that an informative osteopathic, person-centered dynamic assessment could complement holistic and comprehensive evaluations. Furthermore, this approach has the potential to serve as a shared model within an interprofessional context, involving osteopaths, manual therapists, physiotherapists, bodyworkers, and movement specialists. For these reasons, we propose an interprofessional dynamic functional assessment approach for evaluating neuromyofascial movement patterns. This article also outlines a research roadmap for validating the proposed clinical assessment tool.

## 2. Methods

The reporting methodology applied in the current perspective paper adheres to accepted standards for producing a commentary [27]. A working committee of professionals [28] with at least 10,000 h of experience in clinical osteopathic practice (FB, LA, GC, GD’A, RZP, MT, and CL), manual therapy, bodywork and sport science practice (RS, AMV), education, and scientific research (FB, LA, GC, GD’A, RZP, MT, CLRS, and AMV) produced the theoretical underpinning for the current commentary. Further, the framework resulted from a brainstorming process based on the best available evidence and clinical observation.

## 3. Results

The existing literature from the past two years on patient active–participative approaches [22] has been reviewed in the context of the neuroaesthetic enactive paradigm to propose an initial conceptual and operational framework [5]. For the sake of readability, a glossary of the relevant terms of the mentioned conceptual framework and related clinical model are reported in Table 1.

Selected findings of the literature search were grouped and are reported in order of relevance in seven relevant sections as follows: Section 3.1. History and Evolution of Patient Active–Participative Approaches in the Osteopathic Field; Section 3.2. Movement Organization and Motor Abilities: The Role of the Neuromyofascial System; Section 3.3. Functional Neuromyofascial Activity: Motion Assessment to Inform Osteopathic Person-Centered Care; Section 3.4. Proposed Functional Neuromyofascial Activity Scoring; Section 3.5. Functional Neuromyofascial Activity: Score Interpretation Proposal; Section 3.6. Functional Neuromyofascial Activity as Patient Active–Participative Osteopathic Approaches; and Section 3.7. Functional Neuromyofascial Activity: Mental Imagery and Metaphors. The results of our analysis are discussed in Section 4.

### 3.1. History and Evolution of Patient Active–Participative Approaches in the Osteopathic Field

Since its inception, osteopathic care has combined patients’ active approaches with specific advice on lifestyle, including a balanced diet, physical exercise, and recreational activities [29,30,31,32,33]. The passive manipulative approaches often required the active participation of the patient, becoming a truly integrated passive–active approach: for example, through strap techniques, the patient was supported by using osteopathic touch to improve body awareness of the areas involved in the execution of static–dynamic contractions and movements guided through the use of exercise straps [34]. Practitioners’ main clinical objective has always been to motivate patients to practice stress management activities and encourage them to dedicate time to themselves, which are some ways to support the integration of body, mind, and spirit [32], even using the origins of mindfulness and hypnosis techniques [35]. Over the years, there has been a progressive increase in the use of passive manipulative approaches within biomedical and biomechanical-oriented frameworks. The osteopaths preferred a practitioner-centered model instead of promoting strategies that actively involve the patient [36]. Nowadays, in the osteopathic context, there are authors promoting the cognitive involvement of the patient with a more psychologically oriented model [37,38,39,40]. A recently published scoping review aimed to appraise the relevant literature on the functions and principles of patient-active osteopathic approaches (PAOAs) and explored a prospective model for selecting the different types of motor, cognitive, and behavioral strategies, highlighting their integration into patient management approaches [22]. The results of the mentioned review provide insights into the biological and psychological mechanisms of PAOA functions, also describing principles of application of active approaches and their integration with hands-on techniques [22]. PAOAs are considered inter-enactive dyadic strategies (i.e., task-oriented gamification to enhance therapeutic alliance and address agency and uncertainty in daily activities using assisted exercise ergonomics and dietary and lifestyle strategies) [22,40]. To use the body as a pivot, and involve the musculoskeletal system as an interoceptive body image and body awareness generator, PAOAs implement integrated mindfulness, mental imagery, work-in strategies, and fascia-oriented exercise principles [22] (i.e., sensory refinement motion, slow and dynamic stretching, and elastic recoil motion [41]). From the origins of contemporary practice, osteopaths emphasize the musculoskeletal system and related movement as a health generator and focus on maintaining the functionality of the fascial system [22].

### 3.2. Movement Organization and Motor Abilities: The Role of the Neuromyofascial System

Human movement and postural changes related to environmental challenges occur through a dynamic interaction of exteroceptive–proprioceptive–interoceptive perceptive systems integrated at a neuromyofascial level [8]. Movement control depends on musculoskeletal and related body systems functionalities, in which fascial system functional properties have a fundamental role [42]. The fascial system consists of the three-dimensional continuum of connective tissues that interpenetrates and surrounds all organs, muscles, bones, and nerve fibers, supporting the body with a functional structure and providing a domain that enables all body systems to operate in an integrated manner [41,43].

The fascial network of fibrous tissue pervades the entire body, and surrounds, supports, suspends, protects, connects, and divides muscular, skeletal, and visceral components of an organism [43]. Fascial tissue is organized in different layers and compartments [44]. The superficial fascia surrounds the entire body with the exception of its orifices. Internal to the superficial layer is the deep, investing, or axial fascia. The axial layer can be depicted as a tube of fascia that surrounds the vertebral column. The myofascial structures of the limbs are invested by and connected to the body’s midline (i.e., axial fascia) via the appendicular fascia. Internal to the axial fascia of the torso are two additional layers: a layer surrounds the neural structures in the posterior midline, named meningeal fascia [44]; the other layer surrounds all body cavities in the anterior midline and is best termed visceral fascia [44]. Fascial compartments create a connected multidirectional network of myofascial continuity and altered local forces (e.g., via muscular contraction), and it might affect the motion of adjacent tissues and myofascial chains [45]. Although further explorations are needed, recent research supports the existence of myofascial connections and their potential role in human movement and musculoskeletal dysfunctions [43]. Results from animal and human cadaveric investigations reinforce the idea of described myofascial chains named superficial back line, front, and back functional [43]; the lateral line and spiral line are supported by moderate evidence [46]. Myofascial chains provide structural and functional fascial connectivity to nearby structures and could have therapeutic applications for manual and physical therapists [43]. Flexible hierarchical movement organization relies on the fascial structure to create functional synergic linkages at different levels and motor abilities [42]. Motor abilities represent the coordination of the neural system (orchestrating postural, metabolic, cardiovascular–respiratory, psychic, and cognitive functions) and physical elements embedded in our bodies to optimize solutions to movement challenges. Motor abilities are economized by the coordinative role of the fascial system over movement self-organization and are characterized by the coordination between body regions during complex motor tasks [42].

At the base of movement organization, there are mechanoreceptors that exist on the fascia where the neurologically and mechanically generated tensions dynamically balance out [42]. Mechanoreceptors are at the base of postural control, the afferent flow of information to the nervous system about the state of the muscles, and the coordinative myofascial pre-activation of muscular contraction sequences specific for synergy and coordination of different body units [42].

Fascial structure/function interdependence allows the human body to follow a gesture, even a complex one, in an economic, harmonious, and fluid way [47]. Fascial system structural integrity participates in biochemical, physiological, and morphological characteristics that distinguish the physical potential of individuals, and their structural prerequisites (i.e., strength, speed, endurance, mobility, and flexibility). Fascial system functionality includes resting tension of tissue in response to stretch mediated by both passive myofascial tone (intrinsic biomechanical and viscoelastic properties of muscle), and active myofascial tone (i.e., electrical activity within muscle cells) [48]. Human resting myofascial tone (HRMT) is defined as the resting tension of the myofascial continuum, intended as a bio-tensegrity system that includes muscles and connective tissues of the myofascial chains [48]. HRMT favors coordination and synergic movement capacity, allowing the sensorimotor ability to regulate motion and enabling the implementation of musculoskeletal activities rapidly, economically, and accurately (i.e., orientation, balance, agility, sense–movement coordination, and reaction). Alterations in HRMT may be involved in the onset of musculoskeletal dysfunctional and painful conditions [49,50,51]. It seems likely to state that a manual approach [52] and exercise [47] aimed at improving sensory refinement, remodeling fascial tissue, and rebalancing a physiological HRMT could improve the symptoms of patients: working on the mechanoreceptors (somatic equilibrium points) will re-establish a correct synergic activation of the subsequent components involved in a complex movement. HRMT represents the basis of the change from a biomechanical perspective on human movement to a neuromyofascial one [8].

Most of the chief complaints presented to bodyworkers and manual therapists occur within elements of the body-wide fascial net, which are loaded beyond their prepared capability [41]. During the overloading of musculoskeletal and related body systems, the fascial system may lose its functional properties (e.g., reduced coordination skills), consequently affecting movement control [42]. Rehabilitative, educative, and preventive strategies may integrate their focus on musculoskeletal health with connective tissue-oriented approaches [42] or fascia-oriented exercises [41].

In the following paragraphs, a clinical osteopathic model to evaluate the fluidity of movement during motor tasks is proposed. A specific focus was placed on the enhancement of body awareness and, consequently, on the coordinative role of the fascial system in movement self-organization and coordination between body regions during dynamic motor tasks.

### 3.3. Functional Neuromyofascial Activity: Motion Assessment to Inform Osteopathic Person-Centered Care

To assess movement competency, rehabilitation professionals and bodyworkers developed the Functional Movement Screen (FMS) [16,17]. Practitioners use FMS to consider and score a patient’s ability to finalize, perform correctly/incorrectly, and symmetrically/asymmetrically seven standardized movements [16,17]. FMS is a movement-based assessment system that assists the healthcare professional or movement expert and the patient/client in assessing an individual’s foundational movement patterns, physical performance, risk of injury, and readiness to return to physical activity after a rehabilitation process [16,17]. The existing assessment strategies [15,16,17,18] inspired the development of functional neuromyofascial activity (FNA), which has different assessment principles and aims.

The presented FNA model is not intended to replace the existing motor skill evaluation models of conditional abilities (i.e., strength, speed, endurance, and joint mobility) and coordination capacities (i.e., orientation, balance, agility, sense–movement coordination, and reaction) universally shared among the health, movement, and sport science professions. FNA aims to test the ability of the individual to control the synergic coordination of body elements in motion that underlie functional movement accomplished without uncertainty [42]. The synergic coordination of human movement is developed on the modularity of the osteomyofascial system and the preferential communication paths between individual parts or anatomical modules that, in case of functional load or overload, are supported by auxiliary or compensatory pathways [53].

FNA assessment allows the operator to identify the local and global compensatory patterns (i.e., body regions vs whole body). Body regions or fascial compartments, associated with functional myofascial chains—characterized by hesitation and lack of movement fluidity—are not considered as the cause of illness but as access points to elicit an improvement in the patient’s body awareness, in the structure of the osteomyofascial system, and the patient’s control of it in relation to interoceptive and environmental stimuli [22]. Two types of combined verbal–tactile cues are implemented to improve the patient’s body awareness: internal and external cues [54]. In using internal cues, an internal focus of attention directs awareness toward the patient’s body and the action process, as it relates to the movement being executed [54] (e.g., guiding movement by using touch and telling a patient to “push through your heels” when performing an inverted “V” FNA is an example of an internal cue). In using external cues, an external focus of attention directs a patient’s attention toward the effect the action will have on the surrounding environment and the movement outcome, as it relates to the exercise being performed [54] (e.g., guiding movement by using touch and telling a patient to “push through the floor” when performing an inverted “V” FNA is an example of an external cue). Internal and external cues aim to address uncertainty in daily activities and agency, thus enhancing an enactive strategy using a manipulative and exercise-integrated approach [22].

The human sensory action cycle is described by the enactive model as driving structural and functional adaptation to better suit the environment [55]. The proprioceptive, interoceptive, and exteroceptive sensory systems help the brain make predictions about its internal and external context and take adaptive measures to sustain health [55]. A discrepancy between the brain predictive model and the environment (i.e., prediction errors) would produce uncertainty in motion and related functions and, in some cases, promote pain [56]. The dyadic osteopath–patient relationship must aim for a realignment between the predictive model and the environment: the updates of prior predictions through a new generative model produce an enhancement of patients’ positive beliefs about the self in relation to the environment and physical activity [57]. The ecological niche is generated through communication, touch, and movement, and should aim to produce a neuroaesthetic enactive experience [5]. This process aims to improve patient agency: musculoskeletal movement free from uncertainty supported by good functionality of the body systems for regulating environmental demands [19].

The FNA, like other assessment procedures proposed in the osteopathic field [5] and the consecutive personalized therapeutic intervention, are meant to act as verbal and nonverbal interactions between the patient and the osteopath to provide a pleasant surprise and a significant prediction error, which will deviate from prior predictions and, therefore, update the patient’s generative model to better fit the patient’s needs and their ecological niche [5,20,57].

FNA could improve embodied human sense-making by action-oriented relational strategies distributed throughout the brain–body environment. FNA should be promoted in a team-spirit-guided patient problem-solving scenario, where challenges can be discussed verbally and using non-verbal touch-based cues promoted by osteopaths and improved in a task-oriented way. Performing the active assistive exercises in a peaceful and fun way and using metaphors to describe the routine allows the information to be processed in the sub-cortical network [5,22]. When performing assisted exercise, the emphasis is on movement rather than engaging a specific muscle. Through exposure to novel or difficult circumstances in a secure setting, participants’ body–brain unity could re-learn adaptation mechanisms [22].

**Figure 1:**
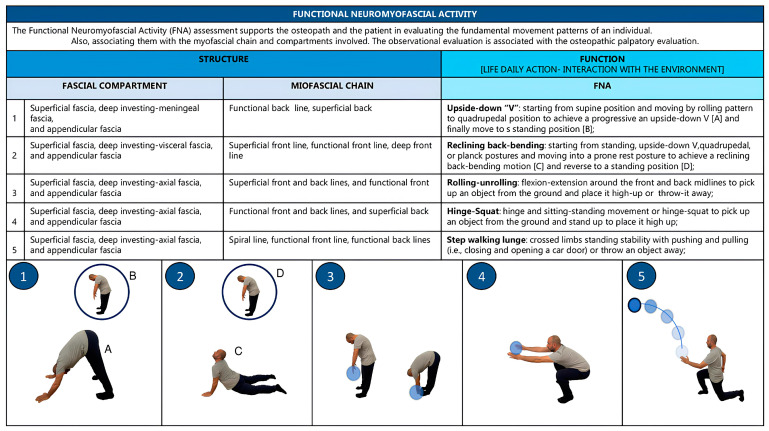
FNA, fascial compartments, and myofascial chains.

The FNA screening system has the purpose of supporting osteopaths and patients in assessing fundamental movement patterns (which include squat, hinge, tilt, lunge, push, pull, twist, walk, and step) and also associating them with the myofascial chains and myofascial compartments and body framework regions and functions involved in patients’ agency. In our proposal, five movements are considered (i.e., rolling–unrolling, squat, step–walking–lunge, upside-down “V”, and reclining back-bending) (Figure 1). Starting postures, movements, and countermovements should be customized according to the patient’s age and clinical condition (i.e., movement progressions or regressions). Osteopaths can request to repeat the FNA (a maximum of three times) until the patient can perform the movement, with attention to using the three characteristic principles of fascia-oriented exercise [41].

In the present perspective paper, we provide an initial proposal that lays the foundation of the FNA framework. The application of FNA assessment could support the osteopath and patient in both symptomatic and non-symptomatic clinical scenarios based on the following assessment guidelines:

- Evaluate functional motor abilities, with coordination and fluidity occurring during the execution of a requested movement to determine motor skills and promptness in carrying out daily actions, from home activities to physical exercise and sports;
- Identify the potential involvement of somatic dysfunction and fascial patterns in regulative functions associated with movement, such as metabolic, cardiorespiratory, neural, cognitive, and psychic functions [19,58];
- Inform the comprehensive assessment of the person in which movement and palpatory findings are considered key elements. The assessment of motor dysfunctions and movement inabilities is refined and informed by an osteopathic palpatory evaluation for somatic dysfunction and fascial patterns. Somatic dysfunction is defined as an alteration in the body functions related to a region of the body framework, showing a lack of movement coordination [19]. The fascial patterns are considered as an altered body function associated with the whole body, showing a lack of movement coordination [19].

The fascial patterns and somatic dysfunctions are not considered to cause general or regional motor dysfunctions and movement inabilities [5]. Conversely, the areas of interest and fascial compartments associated with functional myofascial chains are considered potentially helpful to convey the effects of osteopathic hands-on and hands-off approaches and improve patient functioning based on the following assessment guidelines [59]:

- Evaluate readiness to return to daily activities and physical activity at the end of osteopathic treatment and/or rehabilitation and/or re-education after an injury or surgery;
- Obtain elements concerning the prevention of injuries and the predictability of performance;
- Provide personalized, specific, and functional recommendations for osteopathic treatment and exercise programs;
- Better share with the patients and other health professionals the outcome of the osteopathic assessment of musculoskeletal and associated functions and the aim of the treatment planning for agency and health maintenance.

### 3.4. Proposed Functional Neuromyofascial Activity Scoring

The FNA aims to evaluate the patient’s fluidity in motor abilities. It focuses on the coordinative role of the fascial system over motion self-organization and coordination between body regions during requested functional movements.

**Figure 2:**
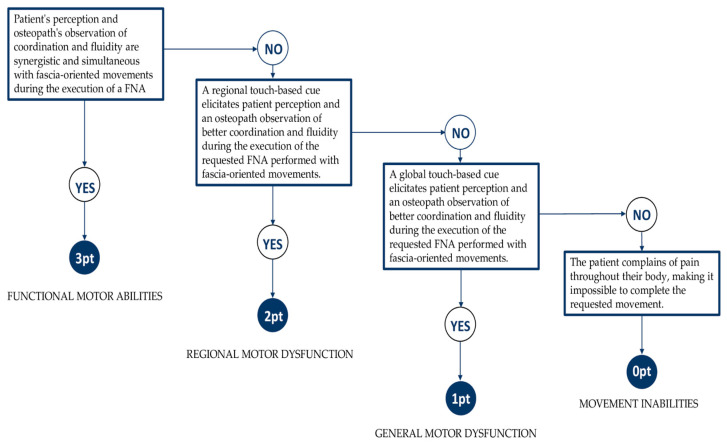
FNA scoring algorithm.

The patients should show coordination and fluidity during the movement execution and repetition, demonstrating the ability to use the principles of fascia-oriented exercise: (a) the patients show proprioceptive awareness and sensory refinement motion and are able to associate evocative mental images with the movement, and share with the practitioner the perception of coordination between body regions during the requested FNA; (b) the patients can perform the FNA with slow dynamic stretching; (c) the patients can return to the neutral position, or a transition to another movement occurs through an impulse with an elastic recoil. As in the FMS [16], the scoring for the FNA consists of four discrete possibilities. The scores range from zero to three, where three is the best possible score. The assessment should collect both patients’ and practitioners’ impressions and perspectives on the FNA performance in order to have a complete and shared point of view. The FNA aims to assess the motor abilities to describe four different levels of functioning (Figure 2):

- A score of three points is given when during the execution of a single FNA the patients show signs of functional motor abilities. The patients share with the osteopath their perception regarding coordination and fluidity of the movement during the execution of requested fascia-oriented movements. There is an observable coordination and fluidity with fascia-oriented motions during the execution of an FNA.
- A score of two is given when during the execution of a single FNA the patients show signs of regional motor dysfunction, lack of coordination and fluidity, and an inability to use principles of fascia-oriented exercise in a specific body area. Only following external and/or internal regional touch-based cues provided by the osteopath (e.g., head, cervical, thoracic, lumbar, sacral, pelvic, lower and upper extremities, rib cage, abdomen, and other regions) is there an emergent patient perception and an osteopath observation of better coordination and fluidity during the execution of the requested FNA performed with fascia-oriented movements.
- A score of one is given when during the execution of a single FNA the patients show signs of general motor dysfunction, lack of coordination and fluidity, and an inability to use principles of fascia-oriented exercise in the whole body. Only following external and/or internal global touch-based cues provided by the osteopath (e.g., fascial compartments associated with functional myofascial chains) is there an emergent patient perception and an osteopath observation of better coordination and fluidity during the execution of the requested FNA performed with fascia-oriented movements.
- A score of zero points is given when the patients show movement inabilities and report diffused pain in the body; consequently, it is not possible to finalize the requested movement.

### 3.5. Functional Neuromyofascial Activity: Score Interpretation Proposal

**Figure 3:**
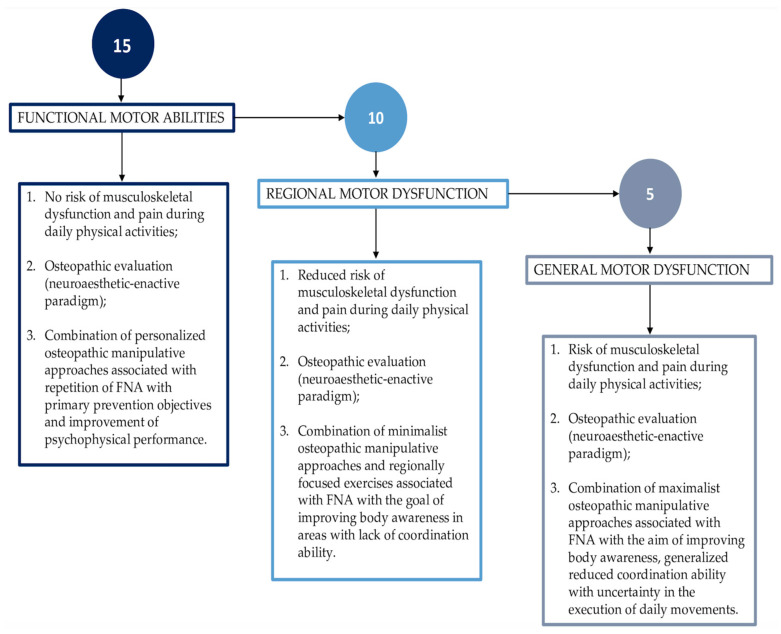
A proposal for score interpretation.

In Figure 3, a proposal for interpreting the FNA score is presented. The evaluation considers five movements, which enables a maximum total score of fifteen points to be obtained.

If the osteopath assigns a maximum score of fifteen to the patients, it indicates that both the patient’s perception and the osteopath’s observations align to support the assessment of functional motor abilities.

The result sustains the hypothesis that there are no signs of risk of musculoskeletal disorder and pain when performing physical daily activities. After the FNA screening, the comprehensive assessment of the person is followed by an osteopathic palpatory evaluation aimed at identifying, in a process of shared decision making with the patient, the type of touch (and possibly the body regions in which to exercise it) that improves the patient’s agency, body functions, and clinical symptoms (if present) [5,19,58,59].

A combination of personalized osteopathic manipulative approaches associated with the repetition of FNAs could be suggested with goals of primary prevention and improvement in psychophysical performance [5,19,22,58,59]. Osteopaths always consider a referral to other health professionals, sports scientists, movement specialists, and bodyworkers.

If the sum of all the five FNA scores reaches a maximum score of ten, then the emergent patient perception and osteopath’s observation support the assessment of regional motor dysfunction. The result sustains the hypothesis that there are signs of a reduced risk of dysfunction and pain when performing physical daily activities. After the FNA screening, the comprehensive assessment of the person is followed by an osteopathic palpatory evaluation aimed at identifying, in a process of shared decision making with the patient, the type of touch (and possibly the body regions in which to exercise it) that improves the execution of the requested FNA. Usually, during the execution (and repetition) of the requested FNA, the patient reports the perception of improved fluidity, applying work and mental imagery principles to synchronize the body regions suggested by the osteopathic touch and related verbal cues.

The presence of somatic dysfunctions detected in a shared decision-making process and associated with body regions guides the osteopath toward the selection of minimalist approaches [5,19,22,58,59]. In addition, the osteopath proposes tailored exercises to improve body awareness in the regions involved in reduced coordination skills [22]. Moreover, osteopaths suggest the repetition of the FNAs following the principles of fascia-oriented exercise to monitor the improvement in the coordinative capacity [22]. Finally, osteopaths promoting therapeutic education always consider a referral to other health professionals, sports scientists, movement specialists, and bodyworkers to improve self-care [22].

If the sum of all five FNA scores reaches a maximum score of five, then the emergent patient perception and osteopath’s observation support the assessment of general motor dysfunction. The result sustains the hypothesis that there are signs of a risk of dysfunction and pain when performing physical daily activities. After the FNA screening, the comprehensive assessment of the person is followed by an osteopathic palpatory evaluation aimed at identifying, in a process of shared decision making with the patient, the type of touch (and possibly the body regions in which to exercise it) that improves the execution of the requested FNA [5,19,22,58,59]. Usually, during the execution (and repetition) of the requested FNA, the patient reports difficulties in applying work and mental imagery principles to synchronize the body regions suggested by the osteopathic touch and related verbal cues. The presence of generalized dysfunctions detected in a shared decision-making process guides the osteopath toward the selection of maximalist approaches [5,19,22,58,59]. In addition, the osteopath proposes the repetition of the FNA following the principles of fascia-oriented exercise to monitor the improvement in motor skills (conditional and coordination capacities) [22]. Osteopaths always consider a referral to other health professionals, sports scientists, movement specialists, and bodyworkers.

FNA represents a simple comprehension tool to assess motor abilities and monitor progressive improvement following an osteopathic care treatment plan. Patients’ understanding of their movement capabilities level, and progress, results in an adherence enhancement, especially in the implementation of self-care strategies.

The scoring levels proposed for the FNA could influence the patients’ exercise mindset and positively affect health outcomes. Such a combined touch–movement-based strategy encourages the patient to obtain a progressive scoring improvement and reach functional ability levels. The practitioners could communicate to the patient that FNA daily repetition is a good exercise, satisfies the recommendations for an active lifestyle and health promotion, and that they can self-assess their motor ability improvements. Moreover, patient mindset (i.e., perceived levels of exercise) could enhance the health benefits of physical activities through the placebo effect [60].

### 3.6. Functional Neuromyofascial Activity as Patient Active–Participative Osteopathic Approaches 

By integrating all the principles of PAOAs, FNA can also be considered a part of osteopathic treatment. PAOAs are task-oriented gamification strategies to enhance therapeutic alliance and address agency and uncertainty in daily activities [22]. PAOAs enforce integrated mindfulness and mental imagery and work in exercise principles to involve the musculoskeletal system as an interoceptive body image and body awareness generator, which are even useful to release stress and restore the body’s homeostasis [22]. PAOAs include assisted active movements based on the principles of fascia-oriented exercise [41] for the patient to better cope with tissue changes and movement hesitation related to the inflammatory effects of sedentary life or to maladaptive priors (e.g., kinesiophobia) often associated with chronic conditions [22]. Fascial-oriented exercise, performed 1–2 times per week for 6–24 months, can affect fascial remodeling and improve movement fluidity [41]. Fascial-oriented exercise implements musculoskeletal movement stimulations like sensory refinement motion, slow-motion stretch, and elastic recoil [41]. The practitioner promotes sensory refinement motion for fascial perceptive mechanoreceptor stimulation [41,59]. It consists of small and complex motions used to increase awareness of perceptually neglected areas of the body, which are also related to interoceptive–proprioceptive sensory alterations. Sensory refinement movement aims to reduce uncertainty by improving the fluidity of the body movement. It was supposed that proprioceptive–motor kinematics experienced during fluid movement leads to enhanced creativity domains: creative generation, cognitive flexibility, and remote associations [61]. Further, the practitioner implements mental imagery and metaphors, showing images to illustrate movement and simplified anatomical–physiological-related elements [22,62].

Slow and dynamic stretching is promoted by melt and unwind movements to make the connective tissue framework more elastic [41]. The preparatory counter movement with tissue elastic recoil mechanism is stimulated through targeted active exercises, in which a preparatory phase increases the elastic tension of the fascial system, followed by a stage where the body releases the weight like a spring [41]. PAOAs are selected and integrated according to the emergent patient pattern occurring from a shared decision-making process [22] achieved with a neuroaesthetic enactive dyadic experience [5] (Table 2). Differently from other bodywork practices, FNA is to be considered a part of assessment and treatment strategies of osteopathic care: based on the osteopathic assessment (i.e., palpation and movement observation informed by FNA screening), personalized manipulative treatment is administered and implemented with a patient active–participative-assisted exercise (i.e., FNA practice)focused on osteopathic palpatory findings (i.e., somatic dysfunction) to improve musculoskeletal movement ability and related body systems activity.

**Figure 4:**
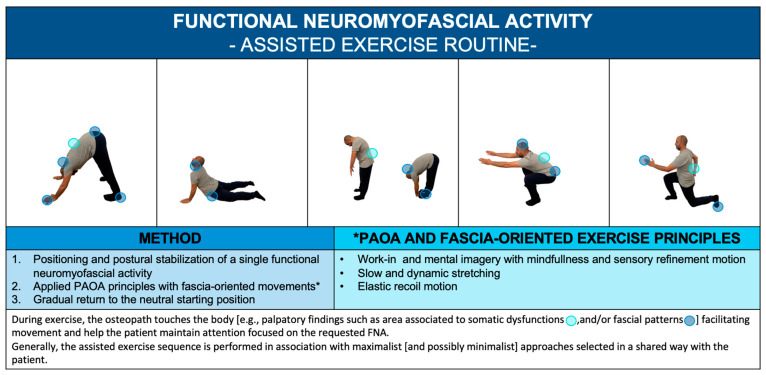
FNA. An example of a patient active–participative osteopathic approach.

Considering the mentioned principles of applications for PAOAs, FNA can be promoted as an assisted exercise (Figure 4; Supplementary Material: Video S1). To reduce the negative impact of sedentary behavior on health, the FNA can be customized for the patient and suggested as exercise snacks: a doable, well-tolerated, and time-efficient approach with isolated ≤1-min bouts of exercise performed periodically throughout the day [63].

Moreover, the practitioner promotes verbal educative content with graphical outlines and visual representations on a screen, a board, or a sheet of paper to improve patient self-care knowledge about FNA, ergonomics, dietary, and lifestyle strategies. For example, a physical activity pyramid can be implemented as a teaching tool to improve functional motor abilities, and a food pyramid could be helpful in providing nutrition details. Participants in these interactions are encouraged to ask questions to negotiate doable tailored cues [22].

**Figure 5:**
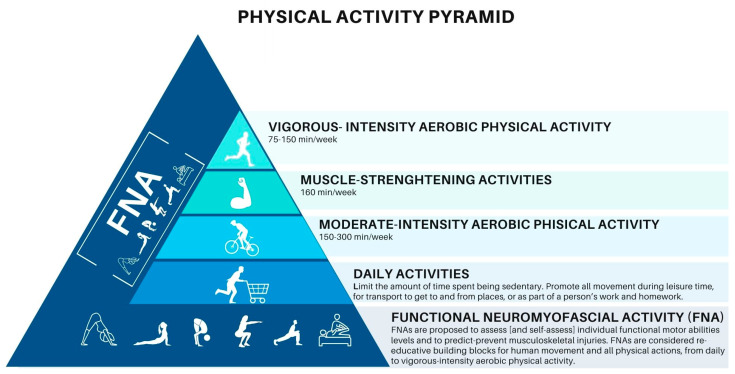
Physical activity pyramid.

WHO guidelines [64,65] report the amount of physical activity needed to be healthy in different age groups and specific populations. The FNA presents an example formalized for adults aged 18–64 years [64,65]. Practitioners implementing the FNA model in treatment planning improve patient adherence to contemporary recommendations for physical activity to maintain good health and well-being and prevent and manage non-communicable diseases [64,65], such as chronic pain related to kinesiophobia (Figure 5).

### 3.7. Functional Neuromyofascial Activity: Mental Imagery and Metaphors

Human actions related to daily life challenges occur through a dynamic integration of perceptive systems’ input mediated by mind–body interactions, i.e., neural processes embodied in the musculoskeletal framework owing to connective tissue, named fascia [8]. The central nervous system organizes movements, tasks, and actions and, therefore, does not organize anatomical activators with clear origin and insertion structures like muscles and ligaments. Movement is orchestrated by the neuromyofascial system promoting functional coordinated patterns to better interact with the environment [8]. The morphological substrate of posture and locomotion occurs through the contribution of the fascial system providing the whole body with widespread architecture for the mechanical forces of transduction and transmission [8].

**Figure 6:**
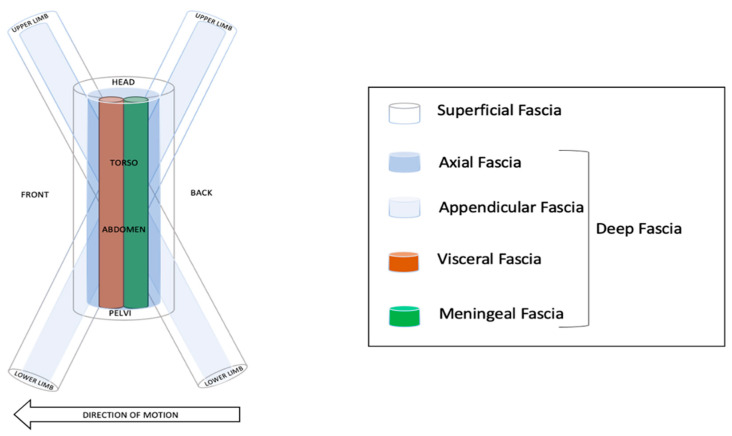
Fascial compartments in motion using FNA.

The fascial system serves as a kind of “connective tissue skeleton”, providing continuity and connectivity to dissipate forces and transmitting biological information in the body framework, being complementary to the anatomical components of skeletal and muscle tissue to promote posture and the locomotion system [44,45]. The fascial system is organized in a series of compartments: “tubes within tubes under the skin” that define the frame of the body (Figure 6). Under the skin, the most external tube is the superficial fascia within which there is a complex tube of axial fascia in the torso and appendicular fascia in the extremities. In the front and back midline to the axial fascia, there are two tubes separated by the vertebral column [44,45]: the meningeal fascia posteriorly and the visceral fascia anteriorly. Retinacula cutis fiber bundles traverse the subcutaneous layer from the dermis to the deep fascia, strengthening the connection between the fascial tubes [44,45].

The fascial network provides protection and lubrication for the elements of the musculoskeletal system and most likely also transmits force transduction during muscle contraction and visceral activities [44,45].

Sedentary life associated with musculoskeletal pain and dysfunction, as well as injuries following athletic and performative overload, are mostly related to fascial tissue changes and are associated with inflammatory processes. Selected movements such as FNA are considered foundational motion patterns and building blocks of human actions and are proposed as screening and re-educative strategies for human movement. Fascia-oriented exercises, such as FNA, performed with an attentive focus on fascial compartment activation during motion can affect fascial remodeling, improve movement abilities, and reduce injuries and pain [41].

**Figure 7:**
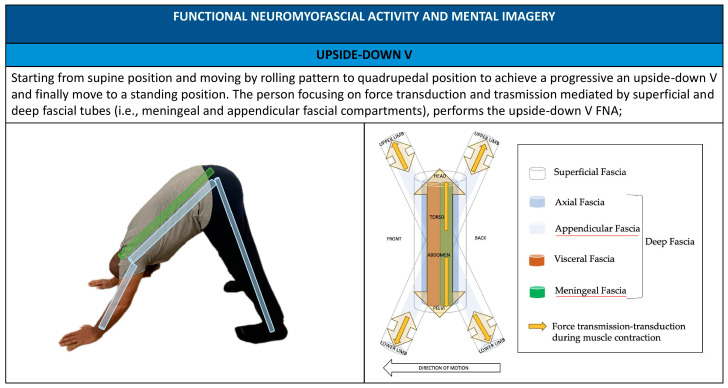
Upside down V FNA; mental imagery and metaphors using FNA.

**Figure 8:**
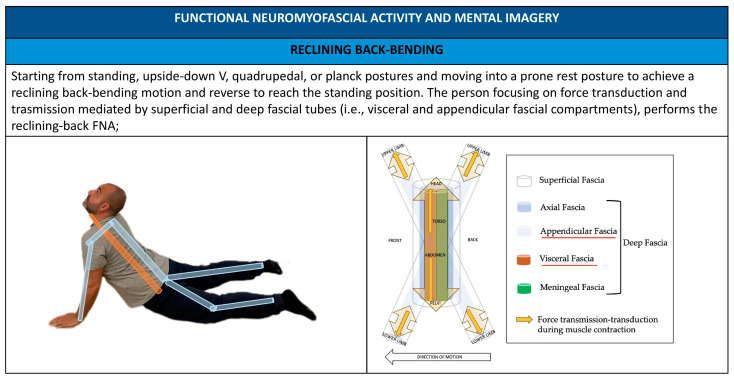
Reclining back-bending FNA; mental imagery and metaphors using FNA.

**Figure 9:**
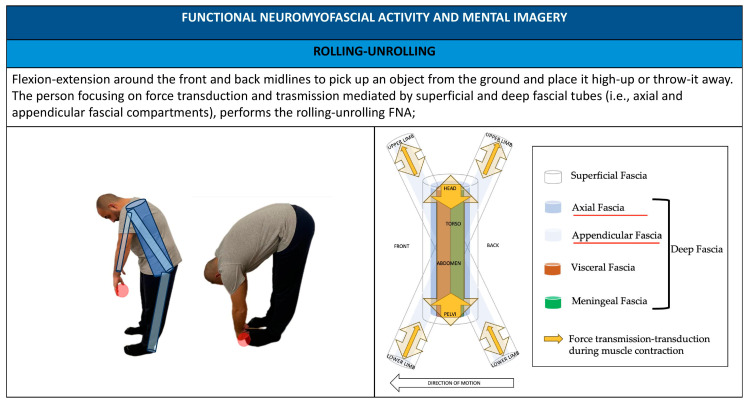
Rolling–unrolling FNA; mental imagery and metaphors using FNA.

**Figure 10:**
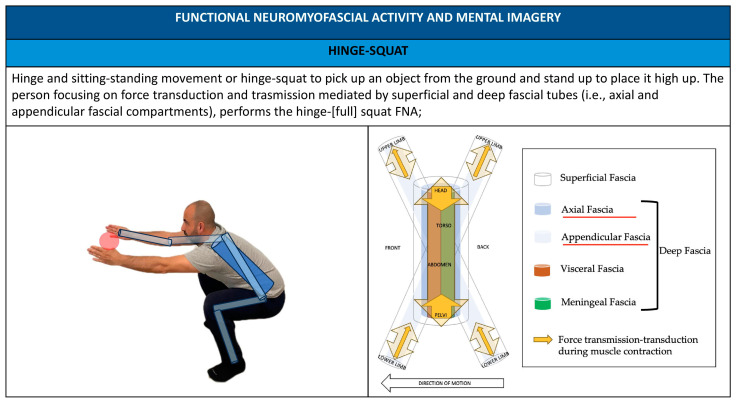
Hinge–squat FNA; mental imagery and metaphors using FNA.

**Figure 11:**
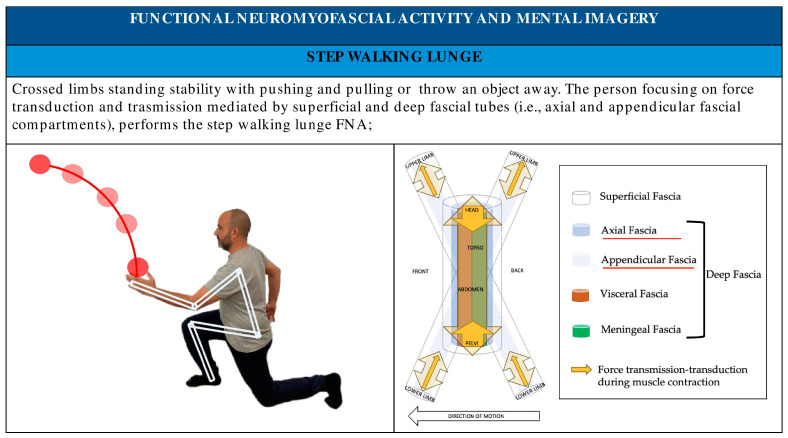
Step–walking–lunge FNA; mental imagery and metaphors using FNA.

Administering FNA practitioners promotes the use of patient mental imagery, implementing anatomical metaphors and showing images to illustrate movement and simplified anatomical–physiological-related elements like fascial compartments (Figure 7, Figure 8, Figure 9, Figure 10 and Figure 11).

**Figure 12:**
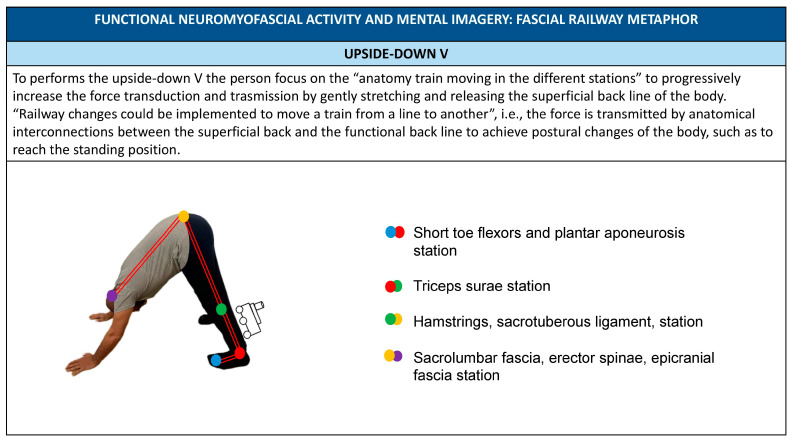
Upside down V FNA and railway metaphors using FNA.

Another example of mental imagery that could be useful to improve patient body awareness and adherence to physical activity is the implementation of a description of the functional transmission of force through the parietal myofascial layer by using the metaphor of a railway network in which anatomical trains move on tracks, representing myofascial chains as the anatomical substrate of the functional coordination of movement [46,66]. In the train anatomy model, the attachments of the individual muscles within the lines are called “stations” to indicate that even if connections to the inner layer occur (formed by the periosteum and ligaments), the transmission of force continues through the fascia beyond the attachment muscle [66]. Different myofascial lines present anatomical interconnections to transmit forces between them and achieve postural changes in the body like railway changes moving a train from one line to another. Through the metaphor of a railway network, it is possible to describe the presence of myofascial connection through the parietal myofascial network [66], such as the superficial back line, front and back functional, lateral line, and spiral line [46] (Figure 12).

## 4. Discussion

The present perspective article proposes an interprofessional functional assessment approach for evaluating neuromyofascial movement patterns moving from passive hands-on approaches to patient-participative approaches, as reported in Table 3 [36,67].

The manuscript provides examples of the significance of such a framework in informing osteopathic person-centered assessments and participative care.

Indeed, focusing on individual activities and encouraging active participation in the movement improvement process is a key aspect of effective therapeutic interventions [68]. Furthermore, patient education, goal setting, tailored exercise programs, empowerment, and motivational techniques are necessary to encourage participation and improve therapeutic outcomes [37,38,39].

An action-oriented assessment and treatment strategy (i.e., FNA) was proposed to be incorporated with comprehensive osteopathic examination and care.

The use of effective communication, verbal and non-verbal (based on touch), allows the usage of the body as a pivot [4], and as an interface with patient body awareness [5]. The gained therapeutic alliance is necessary to finalize a shared decision-making process and plan an individualized treatment integrating manipulative approaches with participatory–educational processes and PAOAs [22]. Such a comprehensive approach, based on a shared decision-making process and integrating hands-on (apparently passive) with hands-off (participative) approaches, could represent a valuable strategy for non-adherent patients to shift from an external locus to an internal locus of control [5]. The article also presents a research roadmap for the validation of the proposed clinical assessment tool. In fact, FNA could represent a simple comprehension tool for patients and practitioners to assess motor abilities and monitor progressive improvement following an osteopathic care treatment plan. Patients’ understanding of their movement capability levels and progress results in an adherence enhancement, especially in the implementation of self-care strategies. Furthermore, FNA could be a valuable transversal interprofessional strategy to improve the collaboration of osteopaths with health professionals or movement specialists and therapeutic exercise promoters, i.e., physiotherapists, occupational therapists, and bodyworkers or yoga–Pilates–fascial-oriented exercise trainers. For example, in a professional collaboration with a physiotherapist expert in fascial manipulation, the osteopath will also consider their putative biomechanical model [69] and describe the myofascial sequences, diagonals, and spirals involved in the patient’s execution of the FNA.

This proposed framework presents some possible limitations. The FNA is proposed to be implemented in the osteopathic management of adult patients. There is a need for adaptations of the model to be used to evaluate physical function in healthy elderly people and in people with musculoskeletal disorders and cognitive function decline. There is a necessity for an elderly population’s personalized adaptive approach to the FNA, especially when implementing doable movement for fragile patients. Another aspect to be discussed concerns the use of the FNA in the pediatric field. Future studies should implement a pediatric adaptive approach to the FNA, considering the use of neural motor developmental stages as decision-making drivers and moderators. Another relevant limitation is that the proposed assessment through the use of FNA has a theoretical basis and, therefore, lacks experimental validation in terms of reliability and validity.

Researchers involved in the future research agenda could consider positive clinical implications in the management of fibromyalgia syndrome [70], chronic low back pain [71], and temporomandibular disorders [72] with manual therapies using interdisciplinary and combined practice approaches [70,71,72], including myofascial techniques [70], effective communication, and pain education [71]. Future studies should aim to enhance the assessment of movement patterns by integrating advanced technologies, such as motion capture, wearable sensors, and computer-assisted analysis, to provide a more accurate and comprehensive evaluation of these patterns [73,74]. Furthermore, it is important to increase collaboration among healthcare professionals from different disciplines, including physiotherapists, bioengineers, and technology experts, in order to develop a holistic approach to the assessment of movement patterns. This approach should involve the development of individualized assessment protocols tailored to the specific needs of patients, along with strategies for patient engagement and education. This will ensure that patients understand the importance of assessing and improving their movement patterns and actively participate in the process.

## 5. Conclusions

The present perspective article highlights the implication of a functional neuromyofascial activity examination and treatment in the context of osteopathic person-centered participative care. We claim that the presented framework could help healthcare practitioners, movement specialists, and bodyworkers in tailoring treatment plans, meeting the patients’ unique needs, and promoting a more effective personalized approach to care.

## Figures and Tables

**Table 1:** Glossary of the relevant terms for neuroaesthetic enactive paradigm [5].

Term	Definition
Neuroaesthetic enactive paradigm	The neuroaesthetic enactive paradigm has been proposed as a framework to describe the neuroaesthetic experiences that occur during osteopathic encounters. The neuroaesthetic enactive paradigm has been proposed as a framework to describe the neuroaesthetic experiences that occur during osteopathic encounters. The diagnostic process and the therapeutic intervention are meant to be verbal and nonverbal exchanges between the patient and the osteopath that will result in a pleasant surprise and a significant prediction error that will defy previous assumptions and update the brain’s generative model. The suggested shared sense decision-making method is based on closeness and nonverbal cues, notably touch, and can be strengthened by using verbal communication with a patient. To avoid nocebo effects and better promote the unique biological and psychological benefits of osteopathic touch, we hypothesize that sharing a pleasant feeling coming from a specifically selected form of executed touch (not another) in a patient’s body location (not another) can convey non-specific effects, such as placebo.
Predictive brain model	Predictive processing refers to any type of processing that incorporates or generates not only information about the past or the present but also about future states of the body or the environment. A positive surprise is evoked in the patient’s priors in the case of the osteopath–patient agreement on pleasant perception as a result of touching an area of interest for both agents, which leads to re-designing predictive models more in line with the ecological–social context in which the patient lives.
Ecological niche in osteopath–patient encounter	An ecological niche is a biopsychosocial context in which there occur interactions between individuals in the ecosystem. The osteopath–patient dyad provides a body–mind state alignment and participates in building an ecological niche in which osteopathic care occurs. The relationship between the osteopath and the patient fills an ecological niche by providing both parties with opportunities to support the patient’s adaptability and ability to reclaim control over their everyday activities, improving their health and well-being.
Therapeutic alliance in osteopathic care	The collaborative relationship between the patient and the healthcare professional, known as a therapeutic alliance, entails a connection between the two individuals as well as a shared awareness of the objectives of treatment and the range of therapeutic tasks and interventions. A combination of effective therapeutic alliance and musculoskeletal approaches (such as osteopathic care) could influence patients’ functions and agency, resulting in the adaptation and restoration of new body narratives and predictive models.

**Table 2:** Patient active–participative osteopathic approaches (PAOAs): selection and integration into treatment processes.

**Assessment**	Multidimensional aspects of patient health (ability to carry out daily activities, familiar symptoms, comparative signs, functional objective examination, and symptom-based objective examination); FNA; Palpatory findings; Emergent patient pattern occurring from a shared decision-making process achieved with a neuroaesthetic enactive dyadic (osteopathic/patient) multi-perceptive experience.
**Osteopathic manipulative treatment**	Personalized manipulative treatment based on the patient’s emergent pattern occurring from a shared decision-making process achieved with a neuroaesthetic enactive dyadic (osteopathic/patient) multi-perceptive experience.
**PAOAs**	The practitioner implements verbal educative content with metaphors, graphical outlines, and visual representations to improve, via gamification, patient self-care knowledge about exercise, ergonomics, and dietary and lifestyle strategies. Personalized exercise based on the patient’s emergent pattern occurring from a shared decision-making process is achieved with a neuroaesthetic enactive dyadic (osteopathic/patient) multi-perceptive experience; for example, FNA can be promoted as assisted exercise.
**Re-assessment**	Multidimensional aspects of patient health (ability to carry out daily activities, familiar symptoms, and comparative signs, functional objective examination, and symptom-based objective examination); FNA; Palpatory findings; Emergent patient pattern occurring from a shared decision-making process achieved with a neuroaesthetic enactive dyadic (osteopathic/patient) multi-perceptive experience.

**Table 3:** Research Roadmap.

Type of Study	Aim
Delphi panel and consensus workshop with grounded theory	To generate a sharable outline of an interprofessional model; To develop FNA models adapted for fragile populations and subjects with more delicate physical conditions.
Validity studies	To evaluate face, content, and construct validity, as well as the reliability and predictive capacity of the FNA in clinical practice to define its clinical usefulness.
Observational studies	To assess patient-reported outcomes, clinical outcomes, and patient satisfaction between subjects treated with integrated passive and active strategies rather than an exclusively passive approach; To evaluate the impact of active–participative approaches on motivation levels and goals for physical exercise; To evaluate the impact of active–participative approaches on quality of life, patient-reported outcomes, and patient-reported experience.
Randomized control trials	To research the effectiveness of exposure to FNA-based exercise as active inference; To evaluate the clinical implications of manual therapy with interdisciplinary and combined practice.
Mentorship, continuing professional development, and consensus workshop	To better contextualize the framework in the approaches promoted by different professionals.

## Data Availability

Not applicable.

## Supplementary Materials

The following supporting information can be downloaded at: https://www.mdpi.com/article/10.3390/healthcare11212886/s1, Video S1: Functional neuromyofascial activity in osteopathic clinical practice.
